# Abstracts and Gleanings

**Published:** 1881-11-20

**Authors:** 


### ABSTRACTS AND GLEANINGS.

**Is Guateu Insane.**—A writer(in Medical Record, N. Y.,) says that Guiteau is unquestionably insane, . because: 1st. Guiteau entertained the delusion that if he obtained a certain position in a foreign embassy, a wealthy woman would marry him and endow him with a million dollars, wherewith to maintain his social position. This idea either was a delusion without any basis whatever, or with so frail a basis as to warrant the formation of such a day-dream to no sound mind; at all events, it was an insane conception. 2d. The letter found in his pocket after the assassination, ordering General Sherman to take possession of the jail, aside from its general tenor, which would immediately rouse the suspicion of any experienced alienist, can be interpeted in only one or two ways, either as an attempt to sham insanity on the part of the writer, or as an isolated but distinct proof of actual insanity. 3d. That Guiteau when arraigned for trial was checked in attempting to read a document, which, however, found its way into the columns of the New York Herald, and is an even more characteristically insane document than the letter to General Sherman. 4th. That as far as an almost unanimous testimony of lay witnesses and existing pictures of the assassin permit us to judge, Guiteau has the charaeteristic "insane manner" in a high degree, as well as a suspicious cranial configuration. 5th. That six months before the assassination, Guiteau applied, if I recollect rightly, for a pension, and the examining surgeon made this brief but to day weighty marginal note to the application, now on file: Applicant is insane! 6th. That Guiteau used to have a placard in his room reading "Guiteau, Premier of England," and on different occasions gave other evidences of entertaining the ambitious delusions of what the French term *megalomanie*, and which Marce more happily designated as *folie systematisee*. To be brief, I would say that the just pride of American psychistry, Isaac Ray, would to-day turn around in his grave if he had heard the latter-day members of the association, whose conservative policy he was the ablest defender of, declaring Guiteau sane, in newspaper interviews held after the assassin's hereditary relations, his documents, and other details had become known. When Mr. Blaine, Senator Logan, nay, the deceased victim of the assassin, and an impartial physician had recognized the insanity of Guiteau, and some of those who are supposed to devote their lives to the care and study of the insane unhesitatingly pronounce him sane, the value of the "asylum experience" argument becomes finely illustrated.

The claim made in several journals that Guiteau is legally responsible, because "he knew what he was about," now shows a fear of death, took measures to secure himself against mob violence, etc., sounds rather like the prevailing cant among a certain class of lawyers than the deliberate opinion of scientists. No competent alienist ever attached weight to the apparently and at times actually methodical actions of lunatics, except to consider those very lunatics more dangerous than their weaker-minded comrades, and therefore believed it incumbent to sequestrate such lunatics, before all others, for the safety of society. There is not a scintilla of doubt in my mind, that in Guiteau with his hereditary history, his insane manner, his insane documents and his insane actions were to be committed to any asylum in the land, he would be unhesitatingly admitted as a proper subject for sequestration. The conclusion concerning his legal responsibility following from this is so self-evident that it will require no rebuttal of the "mad dog" argument urged in interviews and even in acknowledged works of merit, on my part, to distinctly set it forth. If the ridiculous dictum were to prevail, that lunatics are to be held responsible for all acts whose nature they appear to understand, our experts might sink to the intellectual level of the jury that pronounced Gosling sane, because he seemed to be well posted on the trial, without any loss to the science of medical jurisprudence. Gosling, in some respects at least, would at that time have behaved less absurdly than Guiteau; within sixteen months thereafter he died of paralytic dementia in a private asylum near Philadelphia!

A thorough study will convince an impartial and competent jury of medical examiners, before whom such a case should be laid, that Guiteau is not only now insane, but that he was never anything else, that his crime was the offspring of insanity, and that in every act he will betray the characteristic feature of querulent monomania. They will also conclude that, inasmuch as his insanity is not the result of his own vices, but based on a defective organization inherited from a diseased ancestry, anything like responsibility, complete or partial, is out of the question.

Not a day passes but adds another to the accumulating evidence in favor of the view here expressed. In a recent issue of the Herald, I find that the assassin has threatened President Arthur that unless he is allowed fees for witnesses, etc., he will publish documents that will ruin the Republican party. What but a complete confession of the logical apparatus can account for such vaporings, evidently made in all seriousness.

It will be a matter of regret if the Guiteau matter ever comes before a jury. The temper of the whole land, and justly so, was never before so much excited against an individual as against this assassin. Sane or insane, the narrow-minded official conducting this trial, whom Judge Hoar has already censured for his intemperate zeal that has carried him on more than one occasion beyond the legal limits in this case, will find "experts" who will be only too willing to chime in with the public against what public prejudice stigmatizes as the "insanity dodge." And while the death by the gallows of a lunatic, and particularly of one presenting the repulsive though morbid features of Guiteau, may be no material loss to the land or his family, yet it is to be feared that his conviction, which, if he is, as I strongly believe, clearly insane, would be nothing but a formal lynch process, will reflect great discredit on American medical jurisprudence.

**Treatment of Intermittent and Remittent Fever.**—This is a very satisfactory part of the discussion of this disease, since, by proper treatment, a large proportion of cases can be readlly cured. And our remedy, as you know, which is most efficient here, is quinine. In my early practice it was the custom before giving quinine to prepare the system for its reception by emetics, purgatives, and blood-letting, and then it was given in very small doses, and only in the hyperpyretic stage. I believe that I was the first in this country to give quinine in large doses. I gave it at once in full doses and at any stage of the disease.

*What Dose Shall We Give?*—To adults I give at once five grains, and repeat the dose every four hours until slight signs of cinchonism are detected. We give it this way to ascertain the tolerance of the individual for the drug. Having discovered this, continue it in full doses till the paroxysm no longer occurs, and in smaller doses for a long time afterward. It is generally given by the mouth, but may be given by the rectum, in double the quantity, by means of enemata. Or if these both be impracticable, by hypodermic injection, in doses about one-half less than by the mouth. But this latter means is apt to lead to abscesses.

There are many other remedies given, but none of them equal in efficiency to quinine. Among them are salicin, strychnia, ferrocyanide of iron, sulphate of berberin, nitric acid and the sulphites.

In effecting a cure, quinia acts as a toxical agent, destroying the low organisms on which the disease depends for its development.

We may sometimes abort a paroxysm by full doses of opium, or by pilocarpine. Very favorable reports of the efficiency of this latter agent have recently been recorded. Any measure that will arrest a paroxysm may effect a cure. During the paroxysms our treatment must be palliative, using with discretion the means that the indications may suggest. Iron should be given for the anæmia attending the disease; nothing will diminish in size the enlarged spleen so speedily as quinia.

We have now to describe a much more dangerous form of this disease, namely, pernicious or congestive intermittent fever, the distinctive feature of which is its fatality. It may terminate in death in a few hours. It is rare in temperate, but frequent in tropical climates.

The only anatomical appearance that distinguishes it from the benign form is the more strongly marked melanotic character of the tissues. We must remember that the pernicious paroxysms may be preceded by several of a benign character.

There are several varieties of pernicious intermittent. We may have simply a condition of profound coma in the cold stage, death taking place before the stage of fever. Or the coma may be accompanied by delirium, vomiting, purging, and convulsions.

Again, there may be simply great prostration, or this may exist with hæmaturia.

In the stage of coma we must use external and internal stimulants, study the indications, and treat accordingly. Our first object is to carry the patient safely through the first paroxysm, then to prevent its recurrence by quinine in full doses, pushed till cinchonism is produced. I give an adult 20 to 30 grains at once, and if a distinct impression is not produced in four hours, I repeat the dose, bearing in mind that the drug may be given in dangerous doses. Keep the patient quiet and warm, and when you expect another paroxysm give an opiate and try the abortive effect of pilocarpine.

*Remittent Fever.*—Simple remittent, often wrongly called bilious fever, is really a variety of intermittent fever. It commences as an intermittent, develops into a remittent, and is followed by an intermittent. The same cause produces both. The difference is that there is a period of remission instead of an intermission, which lasts from three to twenty-four hours or longer, and may have the varieties of the simpler form of intermittent. It may be inaugurated by nausea and vomiting of bile, etc., but it is rare for typhoid symptoms to be developed.

Its anatomical characteristics are the same as those of intermittent. The disease seems to be more frequent in certain climates and certain years than in others. With reference to diagnosis, the remission will serve to distinguish it.

Treatment consists in the prompt administration of quinine.

There is a pernicious remittent fever to which the same remarks apply as to pernicious intermittent.—*Dr. Flint, in Med. Gaz.*

**Treatment of Nasal Catarrh.**—Dr. Griffith, in Medical and Surgical Reporter, says: For a number of years after graduating, I had a dread of treating catarrh, after hearing Dr. Agnew state, in one of his surgical clinics, that he never cured a case in less than six months, and it often required as much as one year. The very idea of the douches, washes, syringes, atomizers, vaporizers, bougies, etc., were repellant. I found the profession gave the subject but little attention; patients generally drifted into the hands of advertising philanthropists, clergymen retired, and otherwise, Indian doctors, east and west, quacks, etc. Perhaps two persons out of every five are afflicted with it, more or less. Thousands of cases have been treated, without cure or relief, by eminent and skillful physicians. The natural result is that post-nasal catarrh is considered incurable by the people.

The cause of failure has been due to a want of a proper understanding in the treatment. Some have treated it altogether locally, while others have relied on constitutional treatment alone. The proper plan is a judicious combination of both.

The trouble and expense attached to the treatments heretofore recommended have deterred both physician and patient from undertaking or undergoing a trial.

I do not propose to enter into a history of its etiology, pathology, etc. Its various forms, perhaps, originate from the same cause, its names, only varying in degrees and localities.

Its treatment is what we are particularly interested in. The cause should be inquired into, and removed, if possible. Observe the condition of the general health, the secretions of the alimentary canal, the kidneys, liver, skin, etc. These should be placed in proper condition if defective. If syphilis or scrofula exists they should be treated accordingly.

It is not necessary to go over the rounds of treatment ordinarily adopted. They are familiar, and are found in most text-books and journals.

Of all the therapeutic agents, I value none more highly than the preparations of petroleum, both locally and constitutionally. Having used pills prepared from condensed petroleum, or petroleum mass, very extensively in bronchial and lung diseases, and having been time and again informed by patients that they cured their catarrh, I have used nothing else as a constitutional remedy for several years, and effected a cure in nearly all my cases, unless the catarrh was kept up by hypertrophy of the nasal mucous membrane. The formula I have usually used in making these pills is as follows—

℞ Petroleum mass.................................. ℥j,

Pulv. cubebæ............................ } aa ℥ss.

Pulv. ipecac comp........................ }

Make pill mass—four-grain pills.

Sig. One three or four times a day.

Use chlorate of potash in making Dover's powders, instead of sulphate. The petroleum and cubebs, by their specific action on the mucous membranes or respiratory tract, heal and soothe; they loosen up the debris which keeps up the irritation. In ozæna their action is palliative; much relief is often obtained; spiculæ of bone are often thrown off and discharged. I formerly used kerosene diluted with milk and used as a spray in an atomizer; this often gave satisfactory results, but since using the pills I have not found it necessary, only in exceptional cases, to resort to local treatment. I have had but a few patients that found it necessary to use the pills more than two or three months; often cases are cured in as many weeks. I now rather solicit business in that line, but formerly I preferred that the patient would go elsewhere. The plan is cheap, convenient, and efficacious. It has this to recommend it, that catarrh is often associated with other diseases of the respiratory apparatus, and no more efficacious treatment is found than the preparation above described. It is my sheet anchor in coughs, colds, asthma, bronchitis, and the best palliative in phthisis pulmonalis. The article by Dr. Strother, in July 2d, 1881, number of the Medical and Surgical Reporter, has brought to my notice many cases and reports of its therapeutic value. Being now located in the oil regions, I will supply samples to the profession, and hope they will report results.

**Acute Rheumetism.**—Among the prevailing diseases, rheumatism, in some of its various forms, is reported by physicians from every section of the country. Some correspondents ask for a remedy that will quickly lessen the temperature in the early stage of the disease. During past years I relied principally upon some one of the special sedatives to control the circulation and prevent the retention of heat, and full doses of salicylate of soda (or salicylic acid) and salicin to lessen oxidation. And the results obtained were quite satisfactory—often completely relieving the rheumatic sufferer in from five to twelve days. But this treatment nearly always required to be supplemented by agents to stimulate secretion and excretion in order to free the patient's organism of the poisonous elements of waste; or else a subsequent attack was almost sure to follow, and especially if the patient was exposed to sudden atmospheric changes. Recently, I have thoroughly tested the efficacy of pilocarpus pinnatifolius, in the incubating stage of acute rheumatism, and obtained speedy and gratifying results. The administration of this drug (jaborandi) should be made before the temperature reaches 102 degrees; and if the patient complains of great soreness of the muscles, with diminished capillary elimination, free draughts of warm diluents should precede the remedy, and the patient's bedroom should be kept at a uniform temperature of about seventy degrees. Fl. ext. pilocarpus pinnatifolius, dose, ʒss; and if free sweating does not result in thirty minutes, repeat with warm diluents at short intervals until sweating ensues, after which the intervals should be lengthened to three hours, if a prolonged action of the drug is desired. However, a repetition is not often needed, as the temperature will usually be lessened one to three degrees in as many hours. A few doses of quinia sulph., or salicylate of soda will complete the cure. Many physicians withhold quinia until the temperature is quite under control, and by relying upon infinitesimal doses of aconite often fail to check the causes of the inflammatory process until the disease passes beyond their control, and requires from four to thirteen weeks for "a full run." The list of cases treated by jaborandi embraces four cases of "sciatica" (with a history of one to four attacks each). These proved very tractable after the "free sweat."—*Chicago Medical Times.*

**Syphilitic Alopecia.**—True syphilitic alopecia may exist under two different conditions, either the specific eruptions extend to the scalp and thus bring about the fall of hair, which is exceptional; or, which is more frequently the case, patients see their hair falling out without the least lesion of the scalp, and without being able to give a certain interpretation of these facts. It is not always easy to tell why certain cases of syphilis bring about, more than others, the fall of the hair; this accident is met with in all forms of syphilis, the benign as well as the malignant. Generally speaking, there are certain cases which, more frequently than others, cause the fall of the hair, and they are such cases as are from the start complicated with anæmia, debility, bad condition, and bad nutrition.

Syphilitic alopecia presents certain peculiar characters. It occurs without the patient experiencing any sensation aside from the fall of the hair. It is not systematic and has no special seat. The hair falls indifferently at all points. However, it may be seen under two aspects a little different—sometimes the hair is shed in a manner almost regular; again, it falls off in patches, when it is termed alopecia areata. In the majority of cases the two forms are associated together.

Syphilitic alopecia possesses another characteristic frequently overlooked, and that is, that it is always temporary. It lasts some months, a year or more, when all the hair sprouts up again, even in cases in which the scalp had been entirely denuded. In the case observed by M. Fournier and cited above, the hair was reproduced very beautiful and thick. So this accident which affects so many patients is of no particular gravity, time and treatment always bringing about a cure.

In certain cases syphilitic alopecia destroys the beard, the eyebrows, and all hair-covered portions of the body. Alopecia of the eyebrow is a symptom which should at once put the physician upon the trial of diagnosing syphilis. It acts precisely as it does upon the head, that is, that sometimes it renders the eyebrow thin, sometimes removes the hair completely, to a greater or less extent. When the eyebrow is discovered broken by a bald line, this single symptom is almost pathognomonic of syphilis. For the baldness, which often attacks the brow, proceeds differently and denudes entirely the superciliary region.

The therapeutic indications to carry out are extremely limited, although it is customary to use all local remedies. These means, indeed, are superfluous, and can only act by stimulating a little the growth of the hair, which commences always at a certain time. The only real remedy is the mercurial treatment; but it should be remembered that the prejudice which attributes the loss of hair to the use of mercury, is one of the most difficult to combat. That idea has existed for four centuries. Fracastor has already combatted this erroneous idea, and now the demonstration of its fallacy can easily be made, for it is by thousands that the number of syphilitic cases can be counted, which have lost the hair without ever having taken mercury, while the patients whose hair grow out while under mercurial treatment are not less numerous.—*Journal de Medicine et de Chirurgie. Nashville Journal of Medicine and Surgery.*

**Treatment of Typhoid Fever by Salicylate of Soda.**—M. Caussidou made a communication to the meeting of the French Association for the Advancement of Science at the Congress of Algiers, which was based on thirty-two cases of typhoid fever treated by salicylate of soda, and in which the rise of the temperature and the influence of this drug on the febrile process had been registered with the greatest care, as attested by numerous tracings shown by the writer. M. Caussidou arrived at the conclusion, in opposition to the facts observed in several wards of the Paris hospitals, that salicylated medication gives larger, more certain, and more permanent effects than refrigeration. M. Caussidou has even been in doubt if, by administering salicylate of soda from the outset of typhoid fever, it would not be possible to limit the duration of the disease to the first week (?), and if, at least it would not be possible to obtain a number of cases belonging to the abortive form. Nevertheless, M. Caussidou does not conceal the dangers of salicylate medication. Like other observers, he has noted dyspnœa, precordial trouble, and exhaustion in patients where the salicylate of soda brought on a two sudden apyrexia. To avoid these objectionable results, he proposes to administer salicylate of soda in fractional doses of one gramme given every two hours, and to stop as soon as the temperature falls below 38° Cent. (100.4° Fahr.) In a complicated case of erysipelas, the salicylic medication was powerless to produce a febrile recrudescence brought on by this complication. M. Herard declared that he had nothing but commendation for the use of antiseptics, such as carbolic and salicylic acid, in the treatment of febrile diseases.—*London Med. Record*, July 15, 1881.—*Medical News and Abstract.*

**Acetic Acid in Small-Pox.**—Dr. D. S. Oliphant, of Toronto, writes to the United States Med. Invest., June 15th, that in 1873, he made note from Medical and Surgical Reporter, as follows: The small-pox making great ravages in the mountain district of Austrian Silesia, the government sent Dr. Roth there to test the use of simple vinegar, which he asserted would destroy the germinal cause of the disease in all cases He declared that the germ was similar in structure and growth to the yeast germ, and could not live in contact with Acetic Acid. The report he made to Government on his return home was a marvelous one, of an almost universal success. As a prophylactic, he ordered two tablespoonfuls of common vinegar, with or without water, one hour after breakfast and towards evening for fourteen days. For half-grown and feeble persons one-half this dose, Fumigate sick chamber twice daily with vinegar evaporated from a heated shovel or plate. Dr. Oliphant gives three cases in which he employed vinegar in small-pox, both as a remedy and prophylatic, with complete success. He says that he called in 1879 on editor of Canada Lancet, suggesting publication of these cases, which was declined for want of sufficient evidence. Traveling in the fall, he found in the Baltimore American a full report of special committee on small-pox to Maryland Medical Society, closing with these remarkable words : Of all the virous methods of prophylaxis and cure which have come under the notice of your committee during the recent epidemics, none have proved so successful as the so called "Vinegar cure. Dr. O. wishes "some of the brethren would try this method of cure and report progress."—*N. Y. Medical Times.*

**Treatment of Epilepsy.**—For the benefit of Dr. Miller, of Eureka Springs, Ark., I give my treatment of epilepsy, which has been quite satisfactory to me, and may prove useful to him and others in like dilemma. I first remove all cause as far as possible, and put my patients in the best condition I can by appropriate treatment, I then put them on the following—

℞ Potassii bromidi.......................... gr. xx,
Ammonii bromidi.......................... gr. x.

Mix. Sig. For one dose, three times a day. At the same time I give 1-100 of a grain of sulphate of atropia three times a day, and continue this treatment for six or eight months, after which stop and give—

℞ Zinci valerinatis.......................... ʒj,

Ext. belladonna.......................... gr. v,

Acidi arseniosi.......................... gr. j,

Ext. gentian.......................... q. s,

Pill No. xxx.

Mix. it. The first week give two pills a day; the second week, three pills a day; and the third week, four pills a day, until all are taken. When I refill this prescription, I double the amount of zinc, and after about one month on this course, I go back to the bromides again for six or eight months, and then change again to the pills, alternating in this way, and continuing the treatment for two or three years. If the patient becomes anæmic from continued use of the bromides, give iron in any convenient form ; I prefer dialyzed iron. The above is the treatment I pursue in epilepsy, and can only hope that it may prove as useful to others as it has been in my hands.—*Dr. Davis Thera. Gazette.*

**For Nervous Diarrhœa**.—I find tepid baths, night and morning, with internal treatment of sub-nit. bismuth and small doses of freshly pulverized nux vomica, followed by bromide of potash, to effect a cure. In treating inflammatory diarrhœa, our first endeavor should be to obtain a clear diagnosis, and as far as possible not be misled by the patient or its mother. I have often, for instance, seen children who not only would tell me that their whole abdomen pained them, but who would flinch and go on in the most dreadful manner, whenever an attempt was made to examine them, while at the same time there would be no evidence of inflammation, either in the stools or record of temperature taken night and morning. Having ascertained that inflammatory diarrhœa really exists, what is the first thing to do? Is it to give mild purgatives and then strong astringents and opium mixture? By no means. Is it to try some highly recommended preparation or some doctor's favorite prescription? I say most emphatically, no. Every case of inflammatory diarrhœa I have seen has some condition or symptom peculiar to itself. What we should do first of all in these cases (and this applies equally well to all forms of diarrhœa) is to attend promptly to the action of the skin. There being more or less early suppression of the cutaneous secretions, the consequence is a great tendency for the skin to become dry, rough and harsh. To relieve this the child should be bathed morning and evening with warm water, and once freely anointed with camphorated oil; care should be taken to see that flannel is worn next the skin, and in very severe cases a flannel bandage kept around the waist. After using the usual remedies given in the first stages of inflammatory diarrhœa, I know of no better preparation, or one that will give such uniform success as Dover's powder, with the addition of chalk and camphor, which combination was first mentioned by Dr. H. D. Vosbourgh, of Lyons, and is made as follows:

| | | |
|---|---|---|
| ℞ | Opii pulv. | aa ℥i. |
| | Ipecac pulv. | |
| | Potass. nit. pulv. | ℥iv, |
| | Pulv. camp. | ℥ii. |
| | Cretæ preparat (English) | |
| | Rad. glycyrrhizæ pulv. | |

The chalk must be carefully ground with the gum camphor, in order to keep it in a perfect powder, and then the other ingredients added. Dose in proportion to age. The camphor not only acts beneficially as a stimulant, but being chiefly eliminated by the skin and bronchial mucous membrane, assists the action of the Dover's powder.

For the chronic stage of inflammatory diarrhœa the fluid extract of Bael, lately prepared from the Bael fruit, will be found to do well and may be given in conjunction with the usual tonics given in these cases. —*Maryland Med. Jour.*

**Eucalyptus Globulus**.—During the summer of 1879, I was called to visit the White Bluff Female Orphanage of 65 little girls, nine of whom were then sick with well-marked symptoms of "Diphtheria" and very bad cases, two having died from the disease before my arrival. I gave with each dose of quinine from 5 to 10 gtt of Fl. Ext. Eucalyptus, (Tilden & Co.'s), the largest children frequently gargling with sulphite soda, eucalyptus and water, the atomizer being used when a child resisted; the preparation of iron used was the Bedford Iron Alum mass. All not yet taken received the same treatment, and out of the whole number of cases thus treated, nineteen in all, none died. This is not stated egotistically, but with the intention of directing the attention of medical men to this subject; for an agent producing such excellent results is certainly worthy of note.

The cause of the infection was traced to a dry brick well over which the wash house had been built, unknown to the inmates, and which had become half filled with putrid water; the first nine cases were those girls who had washed at that time, a positive evidence. Since then I have treated (two in my own family) many cases with like results; in the family of Mr. J. T. R., of five children, one of the worst cases I ever saw, a very obedient and tractable girl,—recovery—the other four escaped.

With such results before us, certainly no one will deny that eucalyptus possesses anticeptic virtues if nothing else; used as a mouth wash it corrects fœtid odors, etc., to go into further details would, however, extend unnecessarily this article which is submitted for your use.

S. F. DUPON, M. D.

—*Monthly Review of Medicine and Pharmacy.*

**New Method of Producing Anæsthesia in the Larynx.**—Prof. Rossback, in the Ann. des Maladies de l'Orille, de Larynx, et des Organes Connexes, Sept., 1881, describes his method, which consists in an attempt to suspend the conductibility of the trunk of the sensory nerve of the larynx so as to produce complete anæesthesia of that organ. The trunk of the sensory branch of the superior larynegal nerve reaches the interior of the larynx by penetrating the thyro-hyoid membrane, below the extremity of the greater horn of the hoyid bone. At this point the nerve trunk is very superficial, and it is very easy, by means of ordinary agents, to destroy its conductivity. The author uses subcutaneous injections of morphia, 0.005 grm. at this point on both sides of the neck. Success was complete. He also found by experiments made on healthy subjects, that the conductivity of this nerve could be suspended by cold. He used for this purpose a Richardson's atomizer, with two jets so arranged that the spray is thrown on both nerves at the same time. A spray of ether served in less than two minutes to render the interior of the larynx entirely insensible of contact with a foreign body. The author thinks that this method might be of use in cases of reflex spasm, where the point of departure is in the interior of the larynx, as well as in painful affections of this organ.—*Medical News and Abstract.*

**Human and Animal Variola.**—Does cow-pox or vaccinia result from the transmission of variola in the cow? In a late work by the President of the Royal College of Veterinary Surgeons, London, reviewed in the N. C. Medical Journal, we find the following:

"The experiments of the noted Commission of the Lyons Society of Medical Sciences are concisely summarized, and will repay a careful reading. The French Academy of Sciences awarded the Montyon prize, value 2,500 francs, to Chauveau, Viennois and Meynet of the Commission. The following we extract from the verdict of the Academy: 'In establishing that vaccinia and variola, notwithstanding the features which assimilate them in animals as man, are, nevertheless, totally independent of each other; that these viruses form two distinct individualities; that the two affections thus constitute two different, immutable species, which cannot be transformed one into the other; that, consequently, to seek to produce vaccinia from variola would be to pursue a dangerous chimera, which would revive all the dangers of inoculation of by-gone days.' "

**Potassium Bromide in Orchitis and Inflamed Breast.**—Dr. J. Grainer observes, in the Virginia Medical Monthly for September, 1881, that when consulted in time, he finds nothing else necessary, either in orchitis or milk breast, but potassium bromide, in five-grain doses three times a day, or smaller doses more frequently repeated. In advanced or complicated cases, he thinks that auxiliary methods should be used, if only as a precaution, or to expedite the cure; but he has never had the bromide to fail him, even when used alone. In orchitis, a suspensory should always be worn. In some of these cases he has seen the disease held in abeyance for weeks, when the patients would persist in the grossest imprudence, in walking and in horseback-riding. He rarely restricts them in diet. Yet even these cases eventually recovered, without suppuration or atrophy. He has had no opportunity to test it in the metastatic orchitis of mumps, but feels sure it will prove as useful as in the ordinary cases, and he expects to find it sufficient in the next epidemic forms of parotiditis he may meet with.—*Med. and Surg. Rep.*

**Defer's Method of Treatment of Simple Hydrocele.**—Dr. Rol, in the Bull. de Ther., praises this method of treatment, of which he gives the following description: The hydrocele is punctured with cannula and trocar, as usual, and evacuated; through the cannula is introduced a sound, on the end of which is fused a little piece of nitrate of silver; the interior of the tunica vaginalis is then rapidly touched at different points with this caustic, when the sound, and after it the cannula, are withdrawn. The results of this mode of treatment are said to be excellent. Notwithstanding the occurrence of a sharp inflammation, lasting five or six days, a cure is generally obtained, not by adhesion of the two surfaces of the tunica vaginalis, but by a simple vital modification of that membrane. The return of the effusion is rare. Defer's operation is thus described as perfectly safe, thoroughly efficacious, and easily performed.—*Med. and Surg. Rep.*

**Male Wet-Nurses.**—The Journal de Seges Femmes, has a notice of a German physician in Pomerania who makes a specialty of supplying wet-nurses. He excites thc secretion of milk not only independently of pregnancy, but in men as well as in women. An applicant for a wet-nurse is always asked whether *male* or *female* is desired. The former is preferred by some families, under the belief that greater vigor is thus imparted to the infants.—*N, Y. Med. Times.*

**Burns.**—S. W. Stockslager, M. D., (in Chicago Medical Journal and Examiner), says: Seeing so many editorials on maternal impressions, brings to mind a case which strongly impressed me with the positiveness of maternal impression.

I was called on May 23, 1881, to attend a multipara, she being healthy in every respect, but that she had burned her wrist some two months before, and that an old lady had told her that her baby would be marked just as she had been burned, that she had better have a doctor, and then, when the baby was born, the doctor could remove the marks, obliterating all traces of them. Therefore I was called. She was crying when I arrived—afraid her baby would be marked. I told her there was no likelihood of that, and to quiet herself, and not cross the bridge until she came to it. But, lo, when the child was born about two hours after, there was the exact counterpart of the burns on the mother. Although the mother's had entirely healed, the infant's looked like a burn done fifteen or twenty minutes afterward. I treated it just as a fresh burn, and it made a complete recovery without any marks remaining.

Does any one need any stronger evidence of the effect of maternal impressions?

**Protective Syphilization.**—It is an established fact that repeated inculcations of an individual with venereal virus will finally make him proof against the disease. The experiments of Boeck and others, disgusting as they were, developed this fact. Starting from that position, there would be nothing unreasonable in the inference that syphilization "in the natural way," pushed to a great extent, may produce a similar protective influence. Indeed, we know that this is true to a certain extent in gonorrhea. Dr. Eldridge's statement of the vigor and healthfulness of the children of Japan, taken in connection with the fact that their parents have almost invariably suffered from syphilis, is in point. Is it not possible, nay probable, that the character of various diseases may be modified in a series of generations by this means? And will not the same explanation account in part at least, for some of the known changes which have taken place in the forms of disease within the period of history? The subject opens a wide and interesting field for investigation.—*Pacific Medical and Surgical Journal.*

THE Camphor Tree has been planted near Los Angeles, California, with every prospect of success. Why not also the cork oak, which is proved by a tree or two to be perfectly adapted to the climate, there being a good sized one in the town of Santa Barbara. It is too slow for American enterprise?—*Gardner's Monthly.*

**Eucalyptol in Albuminuria.**—Dr. Bauer, of St. Louis, reports several cases of Bright's disease, with marked dropsy, cured by Eucalyptol. The patient had suffered from malarial fever, and there was enlargement and sensitiveness of the liver and spleen. The drug was given in doses of five drops in emulsion, gradually increased to fifteen four times a day, with speedy relief from the dropsy, and an entire cure of the disease in ten weeks.—*N. Y. Med. Times.*

**Bicarbonate of So'a in Tonsilitis.**—La Press Med. Belge, July 17, 1881. Dr. Gine, Professor of Clinical Surgery at Madrid, states that bicarbonate of soda, applied topically and repeatedly to the tonsils, is of incontestable efficacy in quinsy. The remedy may be emyloyed by insufflation through a paper tube, or may be applied by the finger, even by the patient himself. Dr. Gine has rapidly cured dozens of cases by this procedure. In no single case was the application entirely without effect; most commonly a cure was obtained in twenty-four hours. Alleviation took place, ordinarily, at once. In none of his cases was it necessary to wait long for relief.

But he especially recommends this remedy in the prodromic period to abort the disease. Dr. Gine considers tonsilotom for enlarged tonsils as an entirely useless oparation, for this affection is always overcome in a relatively short time by the frequent application of bicarbonate of soda.—*St. Louis Clinical Record.*

**Œsophagoscopes.**—At the meeting of the Royal Society of Physicians, of Vienna, held May 6th, Prof. Stork exhibited his improved œsophagoscope. Fastened to the staff which is to be introduced into the pharynx, is a straight metal tube, which takes the place of the former elastic tube. The metal tube consists of three tubes, which fit into each other; these, by means of a screw arrangement, can be extended so that the tube acquires a length of eight inches and will reach to the cardia.—Wein. Medizin Wochenchr, May 14. Dr. Morrell Mackenzie has invented an œsophagoscope with which a person can view the lining membrane of the œsophagus and possibly even catch a glimpse of the stomach. The œsophageal part consists of two parallel bars, which, after introduction, are opened by an arrangement at the handle and rings separating the bars. A laryngoscopic mirror is attached to the end of the handle.—*Brit. Med. Journal.*

**Chorea of Childhood.**—Dr. S. W. Mitchell, (in Chicago Medical Journal and Examiner,) says: Stormy weather, spring time and schools influence the disease, which has a tendency to recur. Puberty is causative also. This is essentially a disease of cities, and is rare in the Negro. Arsnic is the best remedy.

Habit Chorea—Also a disease of childhood—consists in rapid and repeated twitching of some muscles; rapid winking, shrugging of the shoulder, etc. Most common in girls from seven to fourteen years of age. There has been some fall from the plane of health. A cause cannot always be detected. The child's restraining power is not well exercised. Good and careful diet, light gymnastics, no school, gentle aperients, full doses of arsenic, form the best treatment.

IT is not so very long since a suggestion made by the surgeon Carl Theodor Warren to remove cancer of the stomach was looked upon as "a beautiful dream of youth." However, Prof. Czerny demonstrated practically, about four years ago, that a person can continue to live after the whole stomach has been removed. He cut out the entire stomach, and stitched the œsophagus to the intestines, and the digestive functions were carried on very well and the patient had good health.—*Edinb. Med. Jour.*, May, 1881.

**Quinine Elimination and Absorption.**—Lepidi-Chioti, (Press Medicale Belge,) after a series of experiments on this subject, comes to the following conclusions: That quinine is certainly not eliminated by the saliva or by the perspiration. It is not absorbed after frictions on the skin. When administered hypodermically it appears in the course of some thirteen to fifteen minutes in the urine, and if the prima viæ be in a good condition it is to be found there in from fifteen to eighteen minutes after being administered by the mouth. When an enema containing quinine has been given it is to be detected in the urine within forty minutes thereafter.—*Chicago Medical Record.*

**Tartrate of Quinoline.**—Dr. Donath said that, as regards its physiological action, he found that tartrate of quinoline lowers the temperature of the body materially when introduced into the circulation; in the proportion of 0.2 per cent. it completely prevents the lactic fermentation of milk, the decomposition of urine and gelatine, and the development of bacteria in artificial cultivating-fluids. Therefore, tartrate of quinoline is superior in antiseptic power to sodic, salicylic, carbolic acid, quinine, boracic acid, copper sulphate, and alcohol. In the proportion of 0.4 per cent. it prevents the putrefaction of blood and the curdling of milk. In the proportion of 1 per cent. it completely destroys the coagulability of the blood, and lowers the temperature at which albumen coagulates. . It is decomposed in the system and does not appear in the urine. Therapeutically, quinoline is a very powerful antipyretic in enteric and intermittent fever; it has a striking effect in periodic neuralgia, and is an excellent local antiseptic. It may be given to adults in doses of one to two grams (fifteen to thirty grains) wrapped up in wafers. Children take it easily dissolved in equal parts of syrup and distilled water. It does not cause any unpleasant after-effects, and the absence of giddiness and tinnitus is especially noted.—*London Med. News.*

**Effects of Light on the Skin.**—It has been observed by Dr. Weber that the sensibility of the skin is very much increased in those parts of the body which are always exposed to the light, and this difference has even been measured by that eminent physician. This remarkable fact is especially observable in persons suffering from smallpox, the severity of the disease being visibly augmented if the patient be not confined in a dark room. Dr. Waters states that if the room be so darkened that not a single ray can enter it, the effect is to arrest the disease at the papular or vesicular stage, it never becomes purulent, and the skin between the vesicles is never inflamed or swollen, the liquor sanquinis is not changed into pus, nearly all the pain and itching are absent, and the smell is, if not entirely removed, greatly diminished. Another advantage, important in a therapeutical point of view, is the assistance given to medicine, the absence of light increasing the excretory powers of the skin.—*Druggists' Circular.*

A CASE of acute uticaria from a single three-grain dose of iodide of potash is reported in the British Medical Journal, May 21st. There were none of the usual symptoms of iodism present.—*Marnland Mod. Journal.*

